# Utilizing compassion and collaboration to reduce violence in healthcare settings

**DOI:** 10.1186/s13584-018-0234-z

**Published:** 2018-07-17

**Authors:** Beth A. Lown, Gary S. Setnik

**Affiliations:** 1 The Schwartz Center for Compassionate Healthcare, 100 Cambridge Street, Suite 2100, Boston, MA 02114 USA; 2 Harvard Medical School, Mount Auburn Hospital Department of Medicine, 330 Mount Auburn Street, Cambridge, MA 02138 USA

**Keywords:** Compassion, Patient-centered care, Physician-patient communication, Hospital violence, Emergency department, Patient satisfaction

## Abstract

Violence in healthcare settings is a global problem and violent acts are more likely to occur in emergency departments (EDs). Significant barriers to reporting workplace violence persist among healthcare workers. This, and lack of shared definitions and metrics, increase the difficulty of assessing its prevalence, understanding its causes, and comparing the impact of interventions to reduce its frequency. While risk factors for violence in EDs have been articulated, less is known about how the perspectives of patients and accompanying persons, and their interactions with ED staff may contribute to violence.

We discuss the nature and social context of ED violence and some approaches used to address this problem in the U.S. We argue that perpetrators of violence as well as healthcare staff who experience ED violence suffer when it occurs. While securing safety is paramount, compassionate practices to address this suffering and the social context from which it emerges should be developed and provided for all involved. Collaboration among stakeholders, including patients and family members, may lead to effective approaches to address this problem.

## Workplace violence in healthcare settings is a global problem

Hospitals exist to provide effective, safe and compassionate care for those who are ill, injured, traumatized or suffering. Unfortunately, however, violence against healthcare workers by patients, accompanying persons and visitors is an all too frequent occurrence world-wide within our havens of healing. The implicit challenge posed in the article by Landau et al. is this: Is it possible to effectively diagnose and treat *and* sustain our empathy and compassion towards patients and accompanying persons who threaten or commit violent acts in healthcare settings, while also ensuring the psychological and physical safety of the workforce that serves them? In the study by Landau, et al., the authors approach this question by trying to understand the emotions and perspectives of patients and accompanying persons in emergency departments (EDs) - itself an act of cognitive empathy [1].

To codify these emotions and perspectives, the authors developed a “Negative Feelings Scale.” While scores on this scale were significantly related to all variables measured, a multivariate analysis of aggregate variables showed that among patients, the single significant predictor of negative feelings was low perceived quality of medical care. Among accompanying persons, significant predictors were staff’s general attitudes and quality of ED experience, attitudes toward the patient and low severity of the patient’s medical problem.

We agree with the authors that violence by patients and accompanying persons emerges from the frustration, anger and dissatisfaction they experience in their interactions with ED staff. In addition, ED staff, in the U.S. at least, are also suffering under the weight of myriad organizational and systemic constraints. The larger context of pervasive socioeconomic inequities and the complexity of healthcare systems contribute to a confluence of stressors and negative feelings on all sides that contribute to the emergence of violence. The resultant suffering affects us all. We’ll discuss some of these issues and selected approaches to the immediate question of reducing ED violence.

Verbal assaults and threats by patients or accompanying persons are the most frequent types of violence experienced by ED personnel. Fortunately, hospital-based shootings are infrequent but are more likely to occur in an ED than elsewhere in a hospital [2]. Estimates of physical assault at some point in a healthcare worker’s career range from 8 to 38% globally [3]. Those who spend the most time in direct contact with patients and accompanying persons are targeted most frequently – nurses’ aides and nurses in EDs and geriatric and psychiatric units [4, 5]. Solutions necessarily vary across organizations and regions, as do the sources of violence against healthcare workers. Pertinent to the Israeli study by Landau, et al., one review of violence against nurses internationally suggests that accompanying persons accounted for most cases of physical violence in the Middle East compared with Anglo and European regions where patients themselves were the primary perpetrators [4]. Lack of standardized definitions and reporting systems hinder our understanding of violence in healthcare settings. Data collected by the Bureau of Labor Statistics in the U.S. reflect only violent injuries that result in work absences which fail to reflect the full scope of the problem. However, this data shows that workplace violence is far more common in healthcare settings than any other industry, with 8 cases per 10,000 full-time employees (FTE) for healthcare and social assistance sectors in 2013, compared with fewer than 2 per 10,000 FTE in other large sectors. Eighty percent of serious violence-related injuries in healthcare settings were caused by patients [6]. In a survey of over 7000 U.S. emergency department nurses by the U.S. Emergency Nurses Association, 42.5% reported verbal abuse; 12.1% reported physical violence with or without verbal abuse during a seven-day reporting period [7].

## Risk factors for violence in emergency departments

Many risk factors for workplace violence have been reported. These include infrastructure (inadequate staffing and security, poor lighting and other environmental issues), care delivery problems (long waits, crowding, lack of privacy), and the larger social context. In some countries, the latter includes civil unrest. In the U.S. the presence of guns, gang violence, low socioeconomic status and lack of access to mental and behavioral healthcare are important contributors [8–10]. Patient circumstances are another risk factor, e.g. prisoners in police custody, or patients receiving a devastating diagnosis or prognosis [2, 8]. Patient diagnoses that involve altered mental status due to dementia, delirium, intoxication, and decompensated mental illness are the most frequent characteristics associated with perpetrators of violence in healthcare settings [11].

With the advent of “de-institutionalization” of patients with mental health disorders in the U.S. decades ago, the number of psychiatric inpatient hospital beds has plummeted [12]. The very patients who are most likely to commit violent acts are left “boarding” in EDs for prolonged time periods (varying with insurance type or its lack), awaiting admission or transfer to an inpatient facility [13]. Meanwhile, ED personnel in the U.S. experience intense time-pressure to meet nationally reported quality metrics. This includes, among others, median time from ED arrival to departure, either for admission or to home [14]. These conditions take their toll on healthcare professionals and staff. Daily stressors and violence in the ED coupled with a sense of inability to change them contribute to poor physical and mental health, symptoms of acute stress and post-traumatic stress disorder, high levels of burnout, and intention to leave the organization or one’s profession [15–20]. In fact, ED physicians have the highest rates of burnout among all U.S. physicians, which, by definition, includes emotional exhaustion, sense of inefficacy and treating others like objects rather than human beings [21, 22]. Among ED nurses, post-traumatic stress has been significantly negatively associated with being empathic, providing emotional support, and ability to control emotional reactions [23]. These conditions set the stage for negative interactions with patients who may have altered mental status, and accompanying persons with heightened emotions, including anxiety and frustration.

## Barriers to reporting violence impede solutions

Worldwide, most incidents of workplace violence are not formally reported. Reasons cited include fear of retribution by supervisors, and lack of management accountability. This may reflect an organizational culture that fails to promote patient and professional safety [24]. Many view violence as “part of the job.” Others do not report workplace violence because they believe that many patients who exhibit violent behaviors are not fully in control of themselves due to their underlying conditions [6, 8, 25, 26]. Ample evidence supports this belief, but not necessarily the failure to report violence. Lack of reporting makes it difficult to assess workplace violence prevalence and the effectiveness of interventions to reduce it. Interestingly, in one study, burnout was lower and compassion satisfaction higher among those who reported their tolerance to violence as higher than their coworkers compared with those whose tolerance was similar. However, we know little about the nature of this tolerance [27]. Perhaps, as these authors suggest, tolerance should not imply acceptance, but rather, anticipation that workplace violence will occur and should be accompanied by prevention and preparation.

Patients and accompanying persons who are suffering are generally cared for by dedicated health professionals who are doing their best to serve within departmental, organizational, societal and policy constraints. The toll of suffering is great on both sides of the patient and accompanying persons/professional staff equation when violence occurs. So, we return to the questions raised initially: What interventions will secure our collective safety and psychological wellbeing? And what will enable healthcare professionals to sustain a professional sense of purpose and satisfaction in work, as well as compassion for patients and family members in circumstance that at times imperils them?

## Complex problems require complex approaches

The hospital ED is a microcosm of the larger, inter-related societal, organizational, leadership, management, team and interpersonal contexts in which it is embedded. There are no simple solutions to workplace violence, especially given the need to balance competing values and performance priorities among key stakeholders [28]. Landau et al. propose addressing organizational issues including care quality and processes (e.g. wait-times, lack of privacy), negative attitudes among staff, implementing interpersonal and communication skills training for physicians and nurses, and legislative support for accompanying persons’ involvement in the care of their loved ones in healthcare facilities. Additional approaches have been suggested, although systematic reviews of the impact of potential interventions note the paucity of robust evidence to support any of those studied [29, 30]. Clearly, more rigorous research is needed, but we discuss some interesting approaches below.

International and national guidelines recommend integrated, organizational approaches to workplace violence [9, 31, 32]. The U.S. Occupational Safety and Health Administration offers an organizational “Road Map” to prevent workplace violence based on 5 core elements: management commitment and employee participation; worksite analysis and hazard identification; hazard prevention and control; safety and health training; recordkeeping and program evaluation [33]. Various organizations and companies in the U.S. offer interpersonal and communication skills training (e.g. de-escalation training) to address workplace violence. The Veterans Health Administration (VHA) in the U.S. has developed standardized programs across its facilities nationally that include on-line and in-person training [34, 35]. Each VHA facility has certified trainers for workplace violence prevention and management on staff. Training is tracked in a national training database. These trainings are part of a larger initiative that includes “Disruptive Behavior Committees,” and web-based reporting systems that “red flag” patients who demonstrate disruptive behaviors. The “Patient Red Flag” initiative is controversial among some veterans, and the U.S. Office of the Inspector General has mandated more precise definitions of what constitutes “disruptive behavior” and increased adherence to the program and tracking procedures [36]. We are unaware of any studies that have assessed the outcomes of this initiative.

## Compassion and collaborative practices are necessary and beneficial for all

A “trauma-informed approach” may be helpful for populations that have sustained psychological and physical trauma and abuse. Broadly defined, this includes many patients with mental and behavioral health disorders and victims of violence [37]. This approach shifts the focus from “what’s wrong with you?” to “what happened to you?” [38]. Organizations that adopt this approach teach clinicians and staff about the impact, signs and symptoms of trauma; organizational leaders and managers may integrate this knowledge in policies, procedures and practices. Leadership and management support for the workforce who witness and experience violence directed at them is critical. Compassion practices offered by organizational leaders for clinicians and staff not only benefit them but are also associated with significantly higher patient satisfaction ratings in the U.S. [39]. These practices include recognition for acts of caring and compassion, counseling and pastoral care for staff, and discussion forums for units experiencing conflict, stress and trauma. One such program that provides such forums for multidisciplinary healthcare professionals and staff to discuss the emotional and psychological impact of providing care has been shown to sustain compassion, improve teamwork and decrease psychological distress [40, 41].

The larger societal issues that perpetuate workplace violence are those that affect population health at large – unequal and often inadequate distribution of resources, including access to person-centered, evidence-based and compassionate healthcare, staffing shortages, rising costs of care, and myriad others. Multi-faceted initiatives with participation and collaboration by all stakeholders are needed to address such complex problems, as well as robust methods to study their outcomes. Asking accompanying persons and patients about their experiences and perspectives, as Landau and colleagues suggest, should inform efforts designed to improve the environments in which they receive care. Future innovations may benefit by involving some patients and their loved ones to advise, imagine, and help co-design, with key healthcare stakeholders, the changes necessary to reduce workplace violence [42, 43].

## Conclusions

Landau and colleagues offer important new insights into patients’ and accompanying persons’ negative feelings from which violence emerges. Their data suggests the solutions they propose: addressing care quality, quality of ED experience, improving interpersonal interactions and inclusion. The “Negative Feelings Scale” and other variables examined are significant contributions and provide a potential roadmap for improvement. A factorial analysis of the Negative Feelings Scale might add detail to this roadmap. If two factors are identified (e.g. feelings about received treatment and feelings about staff attitudes), the roadmap may split into pathways to improve perceptions of quality of treatment, and pathways to improve interactions and communication.

Good research questions generate testable offspring: How do patients perceive, describe and judge the predictor, “quality of medical care?” What are the behavioral displays that patients and accompanying persons interpret as patronizing and disrespectful? Can interpersonal/communication training change these? How does “organizational culture,” reflected in the attitudes and behaviors of leadership and management in healthcare settings influence those of staff? What factors reinforce or diminish these attitudes over the trajectory of training and practice? How can we shape these factors to reflect and support our common humanity? Understanding the perceptions and emotions of patients and families is an important piece of this puzzle. Involving patients and families in thinking about solutions may be another. At the same time, we cannot lose sight of the need to support staff for whom episodic violence adds to the daily stress of working in an ED. Violence emerges from and results in suffering. Perhaps by working collaboratively, we can create pathways to preserve our collective safety, health, wellbeing and compassion.
